# Comprehensive Geriatric Assessment: Addressing Unmet Healthcare Needs in Older Adults

**DOI:** 10.3390/healthcare13212715

**Published:** 2025-10-27

**Authors:** Ioanna Dimitriadou, Aikaterini Toska, Sini Eloranta, Susanna Mört, Nina Korsström, Anna Lundberg, Magdalena Häger, Agita Melbarde-Kelmere, Kristaps Circenis, Jekaterina Šteinmiller, Sigrun S. Skuladottir, Ingibjorg Hjaltadóttir, Evangelos C. Fradelos

**Affiliations:** 1 Laboratory of Clinical Nursing, Department of Nursing, University of Thessaly, 41110 Larissa, Greece; ioadimitriadou@uth.gr (I.D.); ktoska@uth.gr (A.T.); 2 Department of Nursing Science, University of Turku, 20014 Turku, Finland; sini.eloranta@turkuamk.fi; 3 Faculty of Health and Wellbeing, Turku University of Applied Sciences, 20520 Turku, Finland; susanna.mort@turkuamk.fi (S.M.); nina.korsstrom@turkuamk.fi (N.K.); 4 Department of Nursing, Åland University of Applied Sciences, 22100 Mariehamn, Finland; annasofia.lundberg@ha.ax (A.L.); magdalena.hager@ha.ax (M.H.); 5 Department of Nursing and Midwifery, Faculty of Health and Sport Sciences, Rīga Stradiņš University, LV-1007 Riga, Latvia; agita.melbarde-kelmere@rsu.lv (A.M.-K.); kristaps.circenis@rsu.lv (K.C.); 6 Department of Nursing, Tallinn Health University of Applied Sciences, 13418 Tallinn, Estonia; jekaterina.steinmiller@ttk.ee; 7 Faculty of Nursing and Midwifery, School of Health Sciences, University of Iceland, 102 Reykjavik, Iceland; sigrunsunna@hi.is (S.S.S.); ingihj@hi.is (I.H.); 8 Landspitali—University Hospital, 102 Reykjavik, Iceland

**Keywords:** Comprehensive Geriatric Assessment, nursing competencies, implementation barriers, older adult, unmet healthcare needs

## Abstract

This narrative review examines the Comprehensive Geriatric Assessment (CGA), a multidisciplinary approach used to evaluate and manage the health of older adults. CGA has been shown to improve functional status, reduce hospital readmissions, delay institutionalization, and lower mortality. Despite these benefits, systematic implementation remains limited. Major barriers include shortages in the workforce and resources, a lack of standardized protocols, and insufficient training in geriatric competencies. These challenges leave many older adults with unmet healthcare needs, particularly in chronic disease management, functional limitations, mental health, and social support. Nurses are well positioned to address these gaps because of their expertise in patient-centered care, care coordination, and chronic disease management. Strengthening geriatric nursing education and integrating CGA into routine nursing practice can improve outcomes for the aging population. Although CGA is often associated with hospital settings, its future lies in broader application. Digital solutions scheduled health assessments, workforce planning, and community- or home-based evaluations can make CGA more accessible. Policymakers, healthcare systems, and educational institutions must work together to develop policies that embed CGA within primary healthcare.

## 1. Introduction

As the population ages, existing healthcare resources are inadequate to meet the complex needs of older adults [1]. One in three people in Europe are predicted to be over the age of 65 by 2070, while the number of individuals over the age of 80 is expected to increase threefold globally [2,3]. Aging demographics, especially frailty and chronically sick older patients with limited mobility, along with all significant contemporary changes, require focused and efficient management.

Comprehensive Geriatric Assessment (CGA) is a multidimensional and interdisciplinary strategy that focuses on the medical, functional, psychological, and human aspects of older individuals aimed at devising effective care strategies [4,5]. Randomized control trials (RCTs), meta-analyses, and health evidence reviews underscore reduced hospital readmission rates, permanent relocation to care facilities, mortality, and enhanced quality of life markers, such as productive functional independence, while preserving mental health [6,7,8,9,10]. CGA has been found to be effective for older individuals in geriatric units but also for frail older adults living at home [11]. CGA is also cost-effective, particularly when combined with optimal resource allocation, reduction in unnecessary interventions, prevention of complication crises, and mitigation of adverse outcomes [12]. A comparison of balancing the costs and benefits accrued illustrates the economic viability of CGA [13].

Although CGA has proven to be effective, its use has been inconsistent, owing to several significant barriers to its implementation. The main barriers include inadequate resources, lack of trained geriatric healthcare professionals, and lack of clear guidance or competencies for performing CGA [14,15]. Facing these issues requires a holistic understanding of the major domains and skills needed for an effective CGA approach and overcoming implementation barriers dominantly [3].

These gaps hinder its widespread adoption and prevent healthcare systems from fully realizing the potential benefits of CGA. Addressing these challenges requires a systematic and holistic understanding of the core domains and skills necessary for effective CGA as well as strategies for overcoming implementation barriers. This narrative review synthesizes current evidence on the unmet needs of the geriatric population and examines the competencies nurses require to deliver CGA effectively. By addressing these objectives, it highlights the critical role of CGA in modernizing geriatric care and meeting the growing demand for evidence-based, person-centered interventions for older adults.

## 2. Methodology

This article was designed as a narrative review, aiming to synthesize and critically discuss the literature on CGA with a particular focus on unmet needs of older adults and nursing competencies.

### 2.1. Literature Search Strategy

The search followed the Preferred Reporting Items for Systematic Reviews and Meta-Analyses (PRISMA) 2020 guidelines. Multiple electronic databases were screened, including PubMed/MEDLINE, CINAHL, Embase, and the Cochrane Library, supplemented by grey literature searches (professional society toolkits, consensus reports, educational curricula). Reference lists of included articles were manually checked to ensure completeness. The search combined keywords and MeSH terms “comprehensive geriatric assessment” OR “CGA” OR “geriatric assessment toolkit” AND ‘’unmet healthcare needs’’ OR “nursing competencies” OR “core competencies” OR “training” OR “education” OR “skills” AND “older adults”. Boolean operators and truncation were used to capture variations across databases. Search terms were tailored to each database’s indexing system (MeSH terms in PubMed, CINAHL Headings, and Emtree terms in Embase) to ensure optimal retrieval of relevant studies while maintaining conceptual consistency across sources. No restrictions were applied on publication year to capture historical and recent developments, though only English-language publications were included.

### 2.2. Inclusion and Exclusion Criteria

Studies were included if they met the following criteria:

- Focused on older adults (≥60 years), their caregivers, or healthcare professionals engaged in geriatric care.
- Examined unmet healthcare needs (medical, functional, psychological, or social) or described the implementation, evaluation, or adaptation of CGA.
- Reported on or recommended competencies, skills, or validated instruments relevant to CGA.
- Were published in English, as peer-reviewed journal articles, consensus statements, curricula, professional guidelines, or validated toolkits.

Exclusion criteria included: non-geriatric populations, studies not reporting CGA-related competencies or unmet needs, or conference abstracts without full-text availability.

### 2.3. Data Analysis

A narrative synthesis approach was used. Studies were coded thematically according to unmet needs (medical, functional, psychological, social) and CGA domains. Recurring patterns were grouped into overarching competency domains (frailty, sarcopenia, functional and mobility assessment, nutrition, cognition, mood, pain and sleep, communication, digital health, cultural competence, leadership, etc.). Special attention was given to the role of nurses, as many studies emphasized their pivotal position in coordinating CGA delivery and addressing unmet needs.

## 3. Results

**Figure 1:**
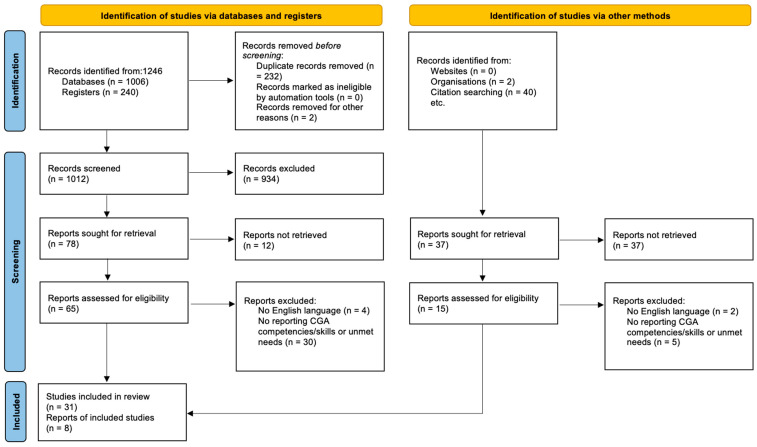
PRISMA flow chart of included studies.

The initial search yielded 2200 records, of which 1874 remained after duplicates were removed. Following title and abstract screening, 152 full-text articles were assessed for eligibility. A total of 39 studies fulfilled the inclusion criteria and were retained for analysis. The selection process is illustrated in the PRISMA flow diagram (Figure 1).

A total of 39 studies were included, covering diverse healthcare settings (hospital, primary care, community, long-term care, and oncology) and targeting frail or multimorbid older adults. The analysis highlighted four major domains of unmet needs: medical, functional, psychological, and social. The included studies encompassed a diverse range of methodologies, including randomized controlled trials, feasibility and pilot studies, observational cohorts, systematic reviews, consensus statements, curricula, guidelines, and toolkits. They were conducted across multiple settings such as primary care, hospital wards, long-term care facilities, and community-based services. The detailed mapping of each study, including design, population, domain of unmet need, intervention, findings, and nursing competencies, is presented in Supplementary Materials Table S1.

### 3.1. Understanding Unmet Needs in Older Adults

Unmet healthcare requirements continue to be a constant issue for older adults, greatly affecting their health results, autonomy, and overall quality of life. Studies show that 23–48% of seniors face at least one unfulfilled healthcare need, with a higher frequency observed in those with lower socioeconomic status, multiple chronic illnesses, and cognitive challenges [16,17,18]. These needs frequently include inadequate medical care, restricted access to preventive services, delays in healthcare delivery, and a lack of support for managing chronic illnesses [19,20].

The consequences of unmet health care needs are substantial. Older adults facing these challenges are at an increased risk of hospitalization, more frequent emergency department visits, and accelerated functional decline. Stein et al. (2019) identified a strong association between unmet healthcare needs and mental health disorders, particularly depression and anxiety [21]. Similarly, Jun Ju et al. (2017) found that individuals with unmet healthcare needs and low economic status experienced a significant decline in health-related quality of life, regardless of the underlying causes [22]. Additionally, a prospective cohort study by Lindström et al. (2020) demonstrated that unmet healthcare needs at baseline were significantly associated with increased all-cause mortality over a five-year period, except in cases of cardiovascular disease, among individuals aged 65 to 80 years [23].

### 3.2. Medical Unmet Needs

Health care deficiencies for older adults are found in medical, functional, psychological, and social areas. Typically, unmet medical needs tend to be associated with chronic illnesses such as hypertension, diabetes, heart disease, and complications related to polypharmacy. A study by Bouldin et al. (2021) noted that almost half of all subjectively declining patients reported not receiving essential medical care [16].

### 3.3. Functional Unmet Needs

Unmet functional needs include difficulties performing activities of daily living (ADLs) such as mobility, bathing, meal preparation, and medication intake. These features further exacerbate dependency, posing an increased risk of entering long-term care [24,25]. Inadequate support for rehabilitation and mobility aids further compounds the problem. Limited access to occupational and physical therapy services can prevent older adults from regaining independence after acute illness or injury. Additionally, environmental barriers such as unsafe housing, poor accessibility, and lack of assistive technologies can turn minor physical limitations into significant disabilities.

### 3.4. Psychological Unmet Needs

Unmet psychological needs are also very important, where undiagnosed and untreated mental health issues, such as depression and cognitive impairment, are prevalent. Evidence suggests many older adults suffering from depression go without sufficient mental health treatment, which in turn exacerbates their physical decline and raises mortality risk [26,27]. A recent meta-analysis found that over one-third of older adults globally experience depression, while Kvalbein-Olsen et al. (2023), found that mental health issues were addressed in only 9.2% of consultations with older patients, and nearly one-third of moderately depressed individuals were unrecognized by their general practitioners, indicating a substantial gap in the recognition and treatment of depression among the older people [28,29]. Moreover, underdiagnosed cognitive disorders, such as dementia, face a lack of timely medical care, resulting in poor long-term outcomes.

### 3.5. Unmet Social Needs

Unmet social needs, including isolation, lack of family or community support and limited access to social resources, can lead to worse outcomes [30]. Older adults who live alone often experience greater difficulties in accessing healthcare due to transportation barriers, financial constraints, and inadequate knowledge of healthcare navigation [31,32]. Inadequate access to community programs, meal services, and caregiver support can exacerbate these challenges, leaving many older adults unable to obtain necessary medical attention or assistance with daily activities. Cultural and language barriers may further limit access to care for minority or immigrant older populations, adding another layer of unmet social need [33,34,35].

### 3.6. Comprehensive Geriatric Assessment

CGA is regarded as a pillar of gerontology, offering a structured, multidimensional approach to evaluating and addressing the complex health care needs of older adults [36]. CGA is a multidisciplinary diagnostic and treatment process that comprehensively assesses an individual’s medical, functional, psychological, and social limitations and leads to the development of comprehensive management plans that enhance overall health outcomes and improve quality of life [36,37]. In addition to these clinical benefits, CGA also contributes to more efficient resource use by reducing unnecessary interventions and avoiding complications, ultimately leading to significant cost savings [9,38,39]. For instance, Singh et al. (2022) found that CGA implementation in primary and municipal care settings reduced emergency visits, hospitalizations, and long-term care admissions [13]

CGA is still being modified for use in various healthcare environments. To maximize the accuracy of functional and social assessments and guarantee that care plans are appropriate for the patient’s environmental context, CGA in the home enables assessments to be carried out in a setting that the patients are accustomed to [40]. Home-based CGA was linked to better overall quality of life, lower rates of hospitalization and death, increased patient satisfaction with care, and improved functional status, according to a recent meta-analysis by Hayes et al. [40]. In a similar vein, CGA models that supported quality of life and preventive care in long-term care facilities were effective in reducing polypharmacy-related measures, improving mental health outcomes, and lowering fall rates [41].

A central strength of CGA is the use of validated instruments across domains to generate actionable care plans. In functional assessment, tools such as the Barthel Index quantify basic self-care abilities, while the Lawton–Brody IADL Scale captures higher-order tasks (e.g., medication management, shopping, food preparation). Lower ADL/IADL scores consistently predict hospitalization, institutionalization, and mortality, and early IADL decline often precedes rapid functional deterioration, supporting targeted rehabilitation and home-safety interventions [42,43]. Physical performance tests—including Timed Up and Go (TUG), usual gait speed, and the 5-times sit-to-stand—provide quick, reproducible markers of mobility impairment and fall risk that correlate with length of stay, subsequent disability, and death [44,45]. Embedding these measures in routine nursing assessment enables timely referral to physiotherapy/occupational therapy and fall-prevention programs.

Frailty assessment adds prognostic precision beyond age and comorbidity. The Clinical Frailty Scale (CFS) offers a rapid 9-point global rating feasible at the bedside and reliably predicts mortality, length of stay, and institutionalization across settings [46,47]. The Edmonton Frail Scale (EFS) provides a multidomain profile (cognition, mood, function, balance, social support, medication, and nutrition) and is practical for nurse-led screening in primary, acute, and long-term care; higher scores are associated with increased adverse events and resource use [48,49,50]. Together, these instruments support nurse-led risk stratification and care coordination, including medication review, exercise prescription, and social support activation.

Given the tight link between muscle health and outcomes, sarcopenia screening and diagnosis should be explicitly integrated within CGA. The SARC-F questionnaire is a fast first-line screen (strength, assistance in walking, rise from a chair, climb stairs, falls); while highly specific, it can miss milder cases [51]. Adding calf circumference (the SARC-CalF variant) improves sensitivity in community and inpatient cohorts. For diagnosis, current consensus (e.g., EWGSOP2) defines probable sarcopenia by low muscle strength—measured with handgrip dynamometry or chair-stand time—and confirms sarcopenia with low muscle quantity/quality (e.g., DXA or bioimpedance). Severity is staged by physical performance (e.g., SPPB or gait speed). Studies link sarcopenia (by these criteria) with increased falls, functional decline, longer hospitalizations, readmissions, and mortality; importantly, nurse-delivered pathways that screen with SARC-F/SARC-CalF, measure grip strength or 5× sit-to-stand, and trigger nutrition plus progressive resistance exercise have demonstrated improvements in function and reduced complications [52].

Nutritional risk intersects both frailty and sarcopenia and should be screened with the Mini Nutritional Assessment (MNA); abnormal results are associated with higher complication rates and poorer recovery, and they guide targeted interventions (oral supplements, meal support, and community services) [1,53]. Psychological and cognitive instruments—including the Geriatric Depression Scale (GDS), MMSE, and MoCA—remain essential, as depression and cognitive impairment amplify disability and impede adherence; routine nurse-led screening improves recognition and aligns care plans with caregiver education and community supports [20,54].

Recent advancements in digital health technologies have transformed CGA delivery. Telemedicine enables remote assessments, expanding access for older adults in rural or underserved areas, whereas artificial intelligence (AI) tools enhance risk prediction and intervention personalization through data-driven insights [55,56]. Mobile health (mHealth) applications are also gaining traction, supporting self-assessments and real-time monitoring in community settings. Pilot programs have demonstrated the potential of mHealth to improve patient engagement and continuity of care, particularly in older adults.

Barriers to widespread CGA implementation are significant and include a shortage in the workforce, know-do gaps, resource constraints, and a lack of standardized protocols, despite the proven benefits of CGA. New models are emerging to address these challenges [57,58]. The task-shifting of CGA components to trained community health workers or nurses has shown promise in low-resource settings. For instance, community health workers in Sub-Saharan Africa have effectively completed basic geriatric assessments which allow for the early detection of health problems. Across the United States, models of collaborative care such as the Program of All-Inclusive Care for the Elderly (PACE) integrate CGA within comprehensive approaches, producing demonstrable improvements in patient outcomes [59,60]. Building on successful examples, such as PACE and municipal-based models in Africa, European countries with rapidly aging populations could benefit from integrating technology-supported CGA models tailored to their healthcare infrastructure.

The future of CGA is person-centered—focusing on the unique needs, preferences and goals of older adults. Including patient-reported outcomes (PROs) in CGA ensures care plans are provided to patients’ which align with their priorities including maintaining independence or enhancing quality of life [61,62]. In addition, computerized techniques of big data analytics and predictive modeling have great potential for the early identification of at-risk individuals and for enabling early interventions to avert adverse outcomes such as hospitalization or institutionalization. These innovations are promising, but successful implementation will have to overcome long-standing barriers to CGA adoption [63].

### 3.7. Barriers to the Effective Implementation of CGA

Although CGA has obvious clinical and financial benefits, several systemic and pragmatic problems have caused inconsistent integration into practice. Even if a lot of data show patient outcome improvement, healthcare systems all around the world struggle with such great operational challenges that they cannot include CGA into their daily operations [36,64]. CGA addresses the many needs that older adults have in many different spheres, as was already mentioned; however, only by addressing major obstacles around workforce and resource availability plus systematic inefficiencies will it capitalize on the possibilities it presents as possible. Dealing with these structural issues is vital; else, care for the older people will remain inadequate, geriatric patients will remain vulnerable, and their many healthcare needs will remain unmet [65].

The scarcity of healthcare professionals with specific knowledge in care and treatment for older people’s needs presents one of the most urgent issues. Many healthcare systems find a serious shortage of qualified professionals able to provide CGA, as the demand for geriatric care increases. Many times, lacking in geriatric-specific competencies, nurses, physicians, and allied health professionals find it difficult to carry out thorough assessments and apply individualized care plans [66,67]. Furthermore, geriatric nursing and geriatrics are underappreciated as a specialty, which results in a workforce shortfall aggravating current provision of healthcare even more [68].

Apart from obstacles related to the workforce, financial restrictions and resource limitations significantly hinder the application of CGA. Successful CGA delivery requires a coordinated multidisciplinary team that includes physicians, nurses, physiotherapists, social workers, and mental health experts. Many healthcare systems, especially in resource- constrained environments, however, lack the infrastructure and financing required to support such cooperative models of treatment [8,69]. Budgetary constraints often lead to understaffing, limited availability of diagnostic tools, and inadequate time allocation for comprehensive evaluation, even in high-income countries. These initial costs continue to hinder widespread adoption of CGA, despite its proven long-term benefits [70,71]. However, recent evidence demonstrates that nurse-led CGA models can be feasibly implemented within the time and resource limitations of primary care, suggesting a scalable and cost-effective approach to expanding access [10].

Without standardized protocols and clinical guidelines for CGA implementation, its heterogeneous application is observed in healthcare settings. Although CGA has been demonstrated to enhance patient outcomes, no consensus exists for a common framework that specifies how assessments should be implemented, documented, and incorporated into subsequent care plans [72,73]. This variability contributes towards a “fragmented” approach to care, whereby certain assessments may be conducted in one place, but do not inform tangible changes in another [74]. There is a lack of structured follow-up processes, leading to the implementation of CGA often being hindered, with many patients not receiving ongoing, coordinated care following the initial assessment [38,75].

More than institutional constraints, policies and frameworks in healthcare systems are more directly responsible for the lack of adoption of CGA that pertains to gaps in communication and infrastructure in both hospital and long-term community services [76,77]. A substantial part of the healthcare system continues to emphasize episodic, acute care rather than moving towards preventive care, such as CGA. Geriatric services are poorly integrated with primary care, community services, and hospitals resulting in fragmented care pathways, which critically misses inter-provider communication [10,78,79]. Moreover, it is common for policy frameworks to not capture reimbursement policies supporting CGA, which results in financial hardships for healthcare institutions with no reimbursable comprehensive assessments. CGA is usually considered a secondary service in the absence of reimbursement incentives, restricting access to many older adults who need these services the most [74,80].

Overcoming the challenges requires specific policy action, higher budget allocations for gerontological and geriatric education, and the creation of uniform protocols for incorporating CGA into standard practice [81]. To address these issues and allow full realization of CGA’s capabilities in health outcome optimization for older patients, there is a need for greater interprofessional communication and collaboration, additional funding, and strategic financial incentives [82,83].

### 3.8. The Role of Nurses in Addressing Unmet Needs Through CGA

Nurses’ diverse clinical and interpersonal competencies make them indispensable for achieving the foundational goals of CGA, particularly in settings where access to geriatric specialist services is limited [84,85]. Nurses play a pivotal role within multidisciplinary teams by working closely with older adults and their families, managing a wide range of chronic conditions, and facilitating person-centered care planning [81,86,87].

Nurses have proven to be extremely beneficial to CGA since they can identify healthcare problems that are often overlooked and taken for granted during interactions with older patients. Because of close contact with patients, nurses provide holistic care which allows them to identify functional decline, cognitive decline, and treatment adherence [88,89]. These proactive measures increase the likelihood of appropriate action, reducing the risk of hospitalization and complications from chronic conditions. Moreover, nursing assessment enhances the possibility of identifying unmet psychological and social factors, such as depression, social withdrawal, social role inactivity, and caregiver role strain, all of which are determinants of health in older adults. Using CGA principles in daily practice helps nurses work towards tailoring individual care plans which address the complex interrelations identified and optimize multi-dimensional wellness [90,91].

**Figure 2:**
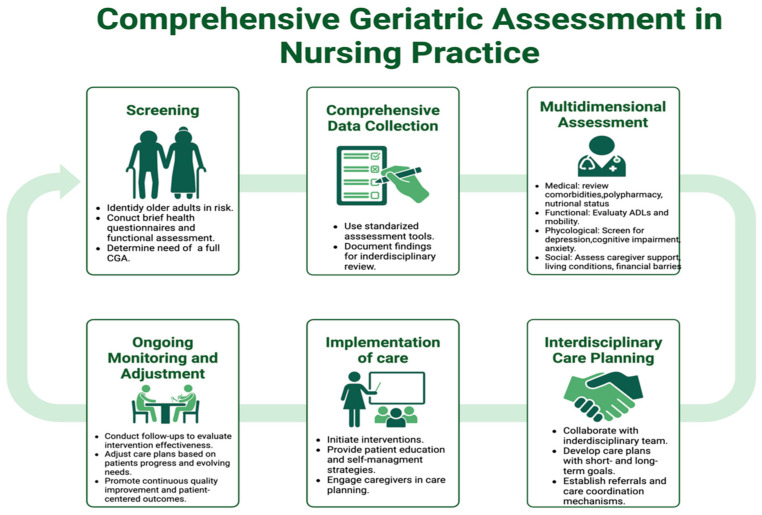
Comprehensive Geriatric Assessment in Nursing Practice. Note: Figure 2 was created by the authors based on the literature synthesized in this review; no third-party materials were used.

Apart from engaging in patient care, nurses play an essential role in integrating interdisciplinary care by ensuring that CGA outcomes are synthesized into workable care plans. Effective CGA implementation requires coordinated teamwork across the healthcare system; however, care fragmentation and siloed pathways continue to impede this process, leading to inadequate interventions [92,93]. Having direct oversight of patient care processes, nurses coordinate bridge gaps in care by capturing vital communication, ensuring that the nurses ‘and patients’ assessments, findings, and multidisciplinary interactions inform appropriate tailored responses. Enhanced clinical outcomes, improved care coordination, and decreased healthcare utilization are hallmark indicators of nurse-directed initiatives, including geriatric case management and community-based CGA [94,95]. As shown in Figure 2, the structured integration of CGA into nursing practice illustrates the integral role of nurses in performing assessments, directing the care coordination processes to ensure continuity of care during transitions throughout the health system.

Despite their critical roles, nurses often face challenges in fully incorporating CGA into their practice. High workloads limit their ability to conduct comprehensive assessments, and gaps in gerontological and geriatric training further hinder effective implementation [96,97]. Dealing with these shortcomings requires focused educational programs, including competency-based systems that provide nurses with the necessary tools for efficient CGA application and specialized geriatric training courses. Maximising the effect of nursing-led geriatric care also depends on institutional support including suitable staffing levels and policy-driven integration of CGA into regular nursing practice [98,99].

Through CGA, nurses are essential in meeting unmet healthcare needs by using their knowledge to deliver comprehensive, patient-centered treatment for the older individuals. Healthcare systems can maximize CGA implementation and improve health outcomes for aging populations by strengthening their involvement in geriatric assessment, enhancing interdisciplinary collaboration, and removing educational obstacles. A key first step toward making sure older adults get thorough, high-quality treatment catered to their changing needs is funding nursing-led CGA projects [100,101]. Currently, the European Big Picture Project (Erasmus+ Alliances for Innovation Programme, https://bigpicture.turkuamk.fi/ (accessed on 20 June 2025)) is developing the Comprehensive Geriatric Assessment Skills Education Program for Nurses. However, further research is needed.

## 4. Discussion

This narrative review synthesizes evidence on the unmet health care needs of older adults and the role of nurses in implementing CGA. Findings suggest that older adults often face overlapping medical, functional, psychological, and social challenges that remain inadequately addressed across health care systems. Integrating CGA into daily practice provides a structured approach to identifying these complex needs and tailoring interventions that promote independence, safety, and quality of life.

Our synthesis shows that CGA is associated with improved outcomes, such as reduced hospitalizations, delayed institutionalization, and reduced mortality rates. Despite these benefits, implementation remains inconsistent, largely due to workforce shortages, limited gerontological education, and the absence of standardized protocols. Strengthening gerontological competencies among nurses, improving interdisciplinary collaboration, and integrating CGA into clinical pathways are essential steps to achieve sustainable integration. Nurses are key to identifying unmet needs, coordinating care, and ensuring that CGA findings are translated into actionable and personalized care plans.

The next phase of CGA hinges on three mutually reinforcing levers—technology, policy, and workforce—working in concert to close persistent care gaps for older adults [102]. Digitally enabled models that blend interoperable electronic records, telemedicine, and AI-driven risk prediction can extend specialist input to underserved areas, flag functional decline early, and personalize care plans in real time [103,104]. While digital innovations expand the reach and efficiency of CGA, they also raise important ethical considerations. Ensuring data privacy and security is essential, particularly when handling sensitive health information. Moreover, equitable access must be safeguarded to avoid widening disparities among older adults with limited digital literacy or resources. Informed consent procedures should be adapted for individuals with cognitive impairment to protect autonomy, and developers must address algorithmic bias in predictive models to prevent inequitable care recommendations.

To make such an innovation routine, national and regional authorities must standardize CGA protocols and link reimbursement to their use, shifting incentives from episodic treatment to proactive, age-sensitive, and team-based care. Clear guidelines and supportive policy frameworks will be essential to embed CGA into mainstream clinical pathways [105,106].

Simultaneously, expanding geriatric competencies across the workforce, through supervised CGA training for nurses, new career pathways in advanced practice, and targeted incentives for all health professions, will help address the ongoing shortage of geriatricians. Education and upskilling are key to making CGA more scalable and sustainable [107,108].

In alignment with global demographic trends, expanding the geriatric workforce is an urgent priority. Implementation strategies should include measures to increase the number of trained gerontologists and geriatric nursing practitioners and clinicians, ensuring adequate capacity to meet the complex needs of an aging population.

Despite promising evidence supporting CGA, inconsistent results exist, particularly in community and low-resource settings. Differences in implementation fidelity, workforce capacity, and patient engagement may contribute to variable effectiveness. Furthermore, limited longitudinal data limit our understanding of the long-term sustainability and cost-effectiveness of CGA across health care systems. Future research should address these gaps through realistic trials and implementation studies that evaluate both successful and unsuccessful models of CGA delivery.

Finally, ongoing research should prioritize municipal- and home-based delivery models. Early evidence suggests that conducting assessments in familiar settings improves patient experience, reduces hospital admissions, and promotes healthier, more independent aging [109]. Together, these strategies position CGA to address the growing and diverse needs of Europe’s aging population. While the discussion draws on European contexts, the principles of CGA are broadly applicable and can be adapted to meet similar challenges in other countries and healthcare systems worldwide.

This review has certain limitations that should be acknowledged. First, as a narrative rather than a systematic review, it may be subject to selection and interpretation bias. To minimize this risk, we followed a structured search strategy based on the PRISMA 2020 framework and applied clear inclusion and exclusion criteria to ensure transparency and reproducibility. Second, only English-language publications were included, which may have led to language bias. However, this decision was made to ensure accurate interpretation of findings and consistency in data synthesis. Third, the included studies varied in design, population, and setting, which limited direct comparison or quantitative synthesis. To address this heterogeneity, findings were organized thematically across major domains of unmet needs and CGA competencies to enable meaningful interpretation. Lastly, while the review primarily focused on the role of nurses in CGA, this focus was intentional to align with the objectives of the European “Big Picture” project and to highlight nursing-led innovations within multidisciplinary geriatric care. Despite these limitations, our structured approach and comprehensive scope enhance the reliability and applicability of the conclusions.

## 5. Conclusions

CGA is an integral component of quality care for older individuals as it helps organize and address the complex health and social needs associated with aging. The advantages of CGA are well documented in the literature, including improved functional outcomes, reduced hospitalizations, and overall cost savings in healthcare systems. Despite these benefits, the widespread adoption of CGA remains limited. This limited adoption stems from gaps in the workforce, scarce healthcare, and infrastructure, as well as a lack of clear implementation guidelines.

Nurses, due to their active role in patient care, interdisciplinary coordination, and chronic disease management, are well positioned to address these barriers directly. Advancing gerontology and geriatric nursing education and integrating CGA strategies into standard nursing duty frameworks will tremendously enhance health outcomes for older adults. With the world facing rapid population aging, policy development, technological appliances, and the creation of new models for delivering care will be vital to support the full potential of CGA. Legislators, healthcare institutions, and educational bodies must collaborate to prioritize CGA and invest in sustainable long-term implementation strategies. A comprehensive, multidisciplinary, and forward-looking healthcare system can better meet the evolving needs of an aging population, ultimately improving the quality of life, promoting healthy aging, and ensuring continuity of care.

## Data Availability

No new data were created or analyzed in this study. Data sharing is not applicable to this article.

## Supplementary Materials

The following supporting information can be downloaded at: https://www.mdpi.com/article/10.3390/healthcare13212715/s1, Table S1. Summary of 39 studies on Comprehensive Geriatric Assessment (CGA), unmet needs and nursing competencies.

## Abbreviations

The following abbreviations are used in this manuscript:

ADLs	Activities of daily living
AI	Artificial intelligence
CGA	Comprehensive Geriatric Assessment
PACE	Program of All-Inclusive Care for the Elderly
PROs	Patient-reported outcomes
RCTs	Randomized Control Trials
