# Supplementary material for: Comprehensive Geriatric Assessment: Addressing Unmet Healthcare Needs in Older Adults
Source: Healthcare (Basel). 2025 Oct 27;13(21):2715. doi: 10.3390/healthcare13212715 (PMC12609822; doi:10.3390/healthcare13212715)
Supplement: Supplementary file 1 [file healthcare-13-02715-s001.zip › healthcare-3916056-supplementary.pdf]

Supplementary Table S1. Summary of 39 studies on Comprehensive Geriatric Assessment (CGA), unmet needs and nursing competencies.

	Author (Year)	Country / Setting	Design & Sample	Domain of Unmet Need	Intervention / Focus	Key findings
1	Garrard et al. (2020) [3]	Europe / Primary care	Systematic review	Medical, social,	CGA in primary care	Improved detection of frailty and unmet needs
2	Rubenstein et al. (1991) [4]	USA / geriatric research	Expert consensus	Medical	Framework for geriatric evaluation	Basis for CGA targeting
3	Veronese et al. (2022) [32]	International	Umbrella review	Medical, functional, psychological, social	CGA outcomes	Improved QoL, reduced mortality
4	Mazya et al. (2019) [6]	Sweden / outpatient	Cohort study	Medical, functional	Outpatient CGA	Reduced frailty progression, mortality
5	Nikolaus et al. (1999) [7]	Germany / hospital + home	RCT	Functional	CGA + home intervention	Improved functional outcomes
6	Ellis et al. (2017) [8,9]	UK / hospital	Systematic review (Cochrane)	Medical, functional	Inpatient CGA	Reduced mortality, institutionalization
7	Stuck et al. (1993) [9]	International	Meta-analysis	Functional, medical	CGA effectiveness	Improved outcomes
8	Xu et al. (2024) [10]	International / hospitals	Systematic review & meta-analysis	Medical, functional	CGA in hospital	Better frailty outcomes
9	Lyndon et al. (2023) [11]	UK / primary care	Feasibility cluster RCT	Functional, social	Nurse-led CGA	Feasible, improved care planning
10	Nord et al. (2022) [12]	Sweden / primary care	Economic evaluation	Medical, functional	Adapted CGA in primary care	Cost-effective
11	Singh et al. (2022) [13]	Multinational	Economic eval.	Medical, functional	CGA hospital-at-home	Reduced ED visits, admissions

12	Mahmoud et al. (2024) [14]	UK / community	Qualitative	Social	CGA in frail older adults	Workforce & workflow gaps
13	Graf et al. (2012) [15]	Switzerland / ED	Observational	Medical	Tools for high-risk older adults	Improved detection
14	Bouldin et al. (2021) [16]	USA / community	Cross-sectional survey	Medical, psychological	Unmet care needs in SCD	50% lacked essential care
15	Herr et al. (2013) [17]	France / community survey	Cross-sectional	Medical,social	Unmet healthcare needs	High prevalence
16	Wang et al. (2024) [18]	China / community	Cross-sectional	Medical,socail	Unmet needs in UHC	Socioeconomic disparities
17	Kowal et al. (2023) [19]	Global / 83 countries	Cross-sectional	Medical,socail	Prevalence estimates	Widespread unmet needs
18	Stein et al. (2019) [21]	Germany / survey	Cohort	Psychological	Unmet needs & depression	Strong association
19	Ju et al. (2017) [22]	Korea / longitudinal	Longitudinal	Psychological	Unmet needs & QoL	Lower QoL
20	Lindström et al. (2020) [23]	Sweden / cohort	Prospective	Medical	Baseline unmet needs	Associated with 5-year mortality
21	Divers et al. (2021)[24]	USA ()	Observational	Functional	Mild functional decline	Identified early difficulties
22	Patrizio et al. (2021) [25]	Not stated	Review	Functional	Assessment methods	Functional tests predictive
23	Huang et al. (2022) [26]	China / nursing homes	National survey	Medical,functional, psychological, social	Unmet needs in LTC	High prevalence
24	Potvin et al. (2011) [27]	Canada / cohort	Longitudinal	Psychological	Anxiety, depression & cognition	Linked to cognitive impairment
25	Cai et al. (2023) [33]	Global	Systematic review	Psychological	Depression prevalence	>1/3 older adults affected

26	Kvalbein-Olsen et al. (2023) [29]	Norway / GP	Cross-sectional	Psychological	Depression recognition	Low detection (9.2%)
27	Pilotto et al. (2017) [34]	International	Review	Medical,functional, psychological, social	3 decades of CGA	Strong evidence across settings
28	Naughton et al. (2023) [35]	Global ageing	Review	Medical,functional, psychological, social	CGA status/future	Need for integration
29	Ellis et al. (2011) [36]	UK / hospital	Meta-analysis RCTs	Medical,functional, psychological, social	CGA in hospital	Reduced adverse outcomes
30	Safari et al. (2023) [37]	UK	Pilot RCT	Medical,functional, psychological, social	Feasibility	Acceptable, promising
31	Ørum et al. (2019) [38]	Denmark / oncology	Cohort follow-up	Medical	CGA in older cancer patients	Lower short-term mortality
32	Fang et al. (2024) [39]	International	Systematic review	Medical,functional, psychological, social	Assessment domains	Key home-based domains
33	Hayes et al. (2025) [40]	International	Meta-analysis	Functional, psychological	Home-based CGA	Reduced admissions, ↑QoL
34	Chadborn et al. (2019) [41]	UK / care homes	Realist review	Medical,functional, psychological, social	CGA in care homes	Improved but variable
35	Katano et al. (2022) [42]	Japan / HF patients	Observational	Functional	Barthel & mortality	ADL score predicted death
36	Ascencio et al. (2022) [44]	Peru / community	Population cohort	Medical,functional	TUG & mortality	Predictive of mortality
37	Richardson (1991) [45]	USA / mobility	Foundational test	Functional	TUG description	Validated test
38	Rockwood et al. (2005) [46]	Canada	Validation study	Functional, psychological	Clinical Frailty Scale	Valid, prognostic
39	Vellas et al. (1999) [53]	International	Tool development	Medical	Mini Nutritional Assessment	Predicts malnutrition, outcomes
